# Investigating the efficiency of lung multi‐disciplinary team meetings—A mixed methods study of eight lung multi‐disciplinary teams

**DOI:** 10.1002/cam4.5730

**Published:** 2023-03-19

**Authors:** Magdalena Zasada, Jenny Harris, Johanna Groothuizen, Eunice Aroyewun, Jeewaka Mendis, Cath Taylor, Madeleine Hewish

**Affiliations:** ^1^ School of Health Sciences University of Surrey Guildford UK; ^2^ Surrey Clinical Trials Unit University of Surrey Guildford UK; ^3^ Royal Surrey NHS Foundation Trust Guildford UK

**Keywords:** behavioural science, cancer management, clinical guidelines, clinical management, lung cancer

## Abstract

**Background:**

Multidisciplinary team meetings (MDTMs), where treatment recommendations are discussed and agreed, are fundamental to effective cancer care. The increasing volume and complexity of caseloads has led to the need to transform MDTM pathways to improve efficiency and allow sufficient time for discussion of complex cases. Understanding of current functioning and inefficiencies is required to inform such transformation.

**Methods:**

A mixed‐methods observational study of all lung cancer MDTMs in one UK cancer network over 12 weeks (*n* = 8 MDTs, 96 MDT meetings). Data were collected on meeting attendance and on each discussed case using a validated MDT tool. Semi‐structured interviews were conducted with a range of MDT members and cancer service managers to gain understanding of perceived influences on the efficiency of MDTMs.

**Results:**

In total, 1671 case discussions were observed. Models of MDT working, including referral and diagnostic pathway management, varied within the network. Attendance was quorate in only 21% of the observed MDTMs, most often lacking palliative care specialists. Over a third (37%) of observed cases were repeat discussions pre‐diagnosis. Treatment recommendations were agreed in 48% of case discussions but deferred for a quarter (24%) of discussed cases, most commonly due to awaiting results. Information about patients' fitness for treatment and/or performance status score was available for 60% of cases discussed overall (30%–75% by MDT). Interviews (*n* = 56) identified addressing clinical and administrative workforce shortages, less reliance on the MDTM for pre‐diagnostic decision‐making and better availability of key clinical information about patients discussed in the MDTM as factors critical to improved MDT function.

**Conclusions:**

Inefficiencies were prevalent in all MDTMs; improvements would require an individualised approach due to the variation in ways of working. Local, regional and national support is needed for lung MDTs to develop their diagnostic workforce and facilities, and clinical and administrative resource.

## Abstract 2

There is considerable variation in MDTM structures and processes, influenced by organisational contextual factors. Inefficient referral and diagnostic pathways were highlighted, causing repeat discussions. Developing the diagnostic workforce and facilities to support implementation of diagnostic pathways will improve efficiency.

## 1 INTRODUCTION

The benefits of multidisciplinary team meetings (MDTMs) (akin to tumour boards) in relation to reducing variation and delivering better patient outcomes in cancer care are internationally recognised.^1^, ^2^, ^3^, ^4^, ^5^ In recent years, as pressure on MDTMs has increased, the focus has shifted to ensuring that MDTMs are efficient and effective at meeting cancer patients' needs. This has been driven by increasing caseloads due to rising cancer incidence,^6^ coupled with increasing complexity in relation to diagnosis and treatment, but without commensurate increases in staffing,^7^ with particular shortages reported in radiology and histopathology in the United Kingdom.^8^ Consequently, MDTMs have become overburdened by routine case discussions and a re‐focus of MDTM discussions on complex cases is being recommended.^3^, ^7^ There is also mounting evidence that the availability of patient information^3^, ^9^, ^10^, ^11^ and investigation results^11^ and the attendance and contribution of a range of MDT members^10^, ^11^ all impact MDTM decision‐making effectiveness.

In the United Kingdom, NHS England has called for streamlining of MDTMs to mitigate these challenges.^12^ This requires a comprehensive understanding of the issues faced currently. The research presented here aimed to better inform MDTM streamlining by conducting an in‐depth investigation of current practice, and barriers to efficient and effective working, in the lung MDTMs within a cancer network. Specific objectives were to:

(1) describe the ‘typical’ characteristics of the MDTMs:

- Attendance and participation;
- Meeting processes and organisation;
- Types of case discussions;
- Types of information available
- Types of recommendations reached in MDTMs

(2) identify key factors likely to improve MDTM working.

## 2 METHODS

### 2.1 Study design and setting

This mixed methods study took place in all eight lung cancer MDTs within a UK regional cancer alliance. This study was reviewed and approved by the Health Research Authority (IRAS 270697) following approval from the NHS London Fulham Research Ethics Committee (ref. 19/LO/1699) and Confidentiality Advisory Group (ref. 19/CAG/0211).

### 2.2 Observational data collection

Non‐participant observational data was collected in virtual lung cancer MDT meetings in each of the eight lung MDTs over a period of 12 consecutive weeks between April 2021 and June 2021.

#### 2.2.1 Development of observational proforma for data collection in MDTMs

Researchers used a modified version of a validated checklist, the MDT QuIC tool,^13^ to capture live observational data for each case discussed in the MDTMs. The tool was initially piloted in over 20 individual MDT meetings, at least twice in each MDT, and including a total of 406 case discussions. During this process, items in the checklist were refined by the research team and clear guidelines written for each item to ensure consistency in use. Inter‐rater agreement was assessed (by having two researchers independently using the tool in MDTMs) and confirmed to be good, with average agreement between raters of 93% (ranging from 84% to 100%).

#### 2.2.2 Data collection

The main lung cancer MDTMs were observed in each hospital. These were defined as meetings where it was intended for all core MDT members to attend, according to the standard set for lung MDT quoracy which specifies the attendance of the following: designated respiratory physician(s); designated thoracic surgeon(s); clinical oncologist; medical oncologist (where the responsibility of chemotherapy is not undertaken by the clinical oncologist core member); imaging specialist; histopathologist; designated cytologist; lung nurse specialist; a core member of the specialist palliative care team; MDT co‐ordinator or secretary.^14^ Data were collected live during the meeting, supplemented with relevant data from the meeting agenda and the Somerset Cancer Register^15^—a web application which holds all cancer patient records across the MDTs (see details of items included in Data S1). Cases discussed at the MDTMs were classified by researchers as pre‐ or post‐diagnostic cases. Pre‐diagnosis cases were defined as patients who at the time of being listed for MDTM discussion had not received a lung cancer diagnosis (some of which may have been listed to have their diagnosis confirmed during that MDTM). Post‐diagnosis cases were defined as patients who at the time of being listed for MDTM discussion already had a confirmed lung cancer diagnosis.

### 2.3 Interviews

Semi‐structured interviews were conducted between April and August 2021 with MDT members and cancer service managers in the eight hospitals by the researchers collecting observational data in each MDT. Participants were asked about their MDT structure and processes and their views were sought about the efficiency and effectiveness of their lung cancer pathway and MDTMs. Interviews were audio‐recorded and transcribed verbatim. Researchers met weekly to discuss emergent findings, as well as sharing interview notes and transcripts from interviews, so that the topic guide could be adapted iteratively in line with emergent findings.

#### 2.3.1 Participants and sampling

Every core MDT member and other regular attenders of each of the eight MDTs participated in the observed MDTMs. Core MDT members and Cancer Services Managers were eligible to be invited for participation in interview(s). Interview participants were purposefully sampled to include members from each of the eight MDTs and represent all core professional groups, as well as each MDT lead clinician (a named core team member with a defined set of responsibilities^14^), where possible. Sampling was undertaken iteratively, with interviewees occasionally suggesting other team members who would be useful to interview, or information provided in interviews lead to the invitation of further team members. Some participants were interviewed twice to gain further insights.

### 2.4 Data analyses and integration

Descriptive statistics (frequencies and percentages for categorical data; or means, standard deviations (SD), medians and interquartile range (IQR) for continuous data) were calculated overall and by MDT to facilitate reporting of the variation in ways of working between MDTs. All statistical analyses were conducted in Stata 16. To protect the anonymity of the participating MDTs only overall percentages are presented (data available on request) and different sets of pseudonyms are used for sets of data presented.

Data relating to MDT ways of working were collated from observation and interviews and cross‐checked for agreement within each MDT. Views relating to the efficiency of MDTMs were analysed thematically, using an inductive approach and constant comparison of data within and between MDTs. Observational and interview data were integrated by using constant comparison between the datasets—cross checking key findings from one dataset with the other, reporting where data were concordant, discordant and/or ‘silent’ on key findings/themes.

## 3 RESULTS

A total of 96 MDTMs were observed including 1671 patient case discussions. This ranged from 4 to 33 case discussions per MDTM (177 to 299 overall from each MDT). Fifty‐six interviews were conducted with MDT members and Cancer Service Managers, lasting between 16 and 51 minutes. Four to eight MDT members were interviewed from each team and at least two members of each core professional group, except palliative care, across the MDTs (Table 1).

**TABLE 1:** Interview participants.

MDT	Interview participants (bold ‐ interviewed twice)
Blue	**MDT lead**, **MDT coordinator, radiologist**, oncologist, pathologist, cancer nurse specialist, cancer services manager
Red	**MDT lead**, respiratory physician, **surgeon**, cancer services manager
Pink	MDT lead, MDT coordinator, pathologist, cancer nurse specialist
Purple	MDT lead, **MDT coordinator**, radiologist, surgeon, oncologist, **cancer services manager**
Orange	**MDT lead, MDT coordinator**, radiologist, **oncologist**
Grey	MDT coordinator, radiologist, pathologist, **cancer nurse specialist**
Green	**MDT lead**, **MDT coordinator**, radiologist, **surgeon**, cancer nurse specialist, cancer services manager
Yellow	MDT lead, MDT coordinator, respiratory physician, radiologist, **cancer services manager**

### 3.1 MDTM attendance

The eight MDTs varied in relation to planned and actual attendance of core team members at the full MDTM. Only four of the MDTs expected all core members to be present for the whole MDTM. In two MDTs, attendance expectations had been adapted so that the pathologist was only expected to attend for a specific part of the meeting where pathology results were discussed. In one MDT, the oncologist and surgeon attended specifically for the oncology and surgery sections of the MDTM, thereby were not always in the meeting together and in another MDT the oncologist, surgeon, pathologist and CNS would leave the MDTM after cases requiring their input were discussed. In 36/96 MDTMs, the attendance of at least one core team member was only ‘partial’ despite being expected to attend the whole meeting. This was particularly marked in the case of surgeons; in 4 of the MDTs where a surgeon was expected to attend the whole meeting, they were only present for the full duration in between 2 and 5 of the 12 observed MDTMs. Partial attendance was also observed for oncologists in four MDTs where they were expected to attend the full MDTM; however, it occurred less frequently than in the case of surgeons and was often the case of them joining the meeting within 15 min of its start.

Of 96 observed MDTMs, 20 were quorate (as defined above) and none of the MDTs achieved quoracy for all 12 observed MDTMs, even when incidences of partial attendance were accounted for. In most cases, this was explained by the absence of palliative care members (at 62 MDTMs). In addition, 6 of the MDTs had 2–3 meetings without the required oncology input, five MDTs held between 1 and 3 MDTMs without a surgeon present, and 1 MDT did not have a pathologist present for any of the 12 observed MDTMs.

### 3.2 MDTM processes

MDTs varied in relation to their MDTM processes including how MDT referrals were triaged for inclusion on the MDTM agenda. Across the eight MDTs, three methods were identified: (1) formal meetings (usually on a set day, sometimes at a set time) between the MDT lead and a radiologist/MDT coordinator/deputy MDT lead (three MDTs); (2) single MDT member completing triage on their own (three MDTs); or (3) ad‐hoc triage where there was no apparent fixed process/time for triage (two MDTs).

The additional forums available for case discussion outside of the full MDTM were also diverse across the MDTs. Only four of the eight MDTs had a separate MDTM for making decisions relating to further diagnostics (a ‘Diagnostic MDT’ as recommended in national guidelines^16^), typically immediately before or after the full MDTM. These meetings varied in scope and attendance between the hospitals that had them, being focussed on early diagnostics/imaging only in three MDTs but including some cases with more advanced diagnostics (radiology and occasionally pathology) in the fourth MDT. However, discussion of some diagnostic cases was observed in full MDTMs in all eight MDTs, regardless of if they had a separate diagnostic MDTM. In most hospitals, there were also other lung cancer‐related meetings such as benign imaging meetings and chest x‐ray meetings.

From the interviews, having insufficient clinical and/or administrative staffing levels were cited by participants as barriers to holding triage or diagnostic meetings. This was also a key factor in achieving quoracy in MDTMs; even MDTs which were adequately staffed often did not have ‘cover’ when a core member was absent/on leave. This was particularly problematic where MDT members were external to the hospital (i.e., employed by and outsourced to the MDT by another organisation) and the MDT would have limited communication with them outside of the meetings.

### 3.3 Types of cases discussed in full MDTMS

**FIGURE 1:**
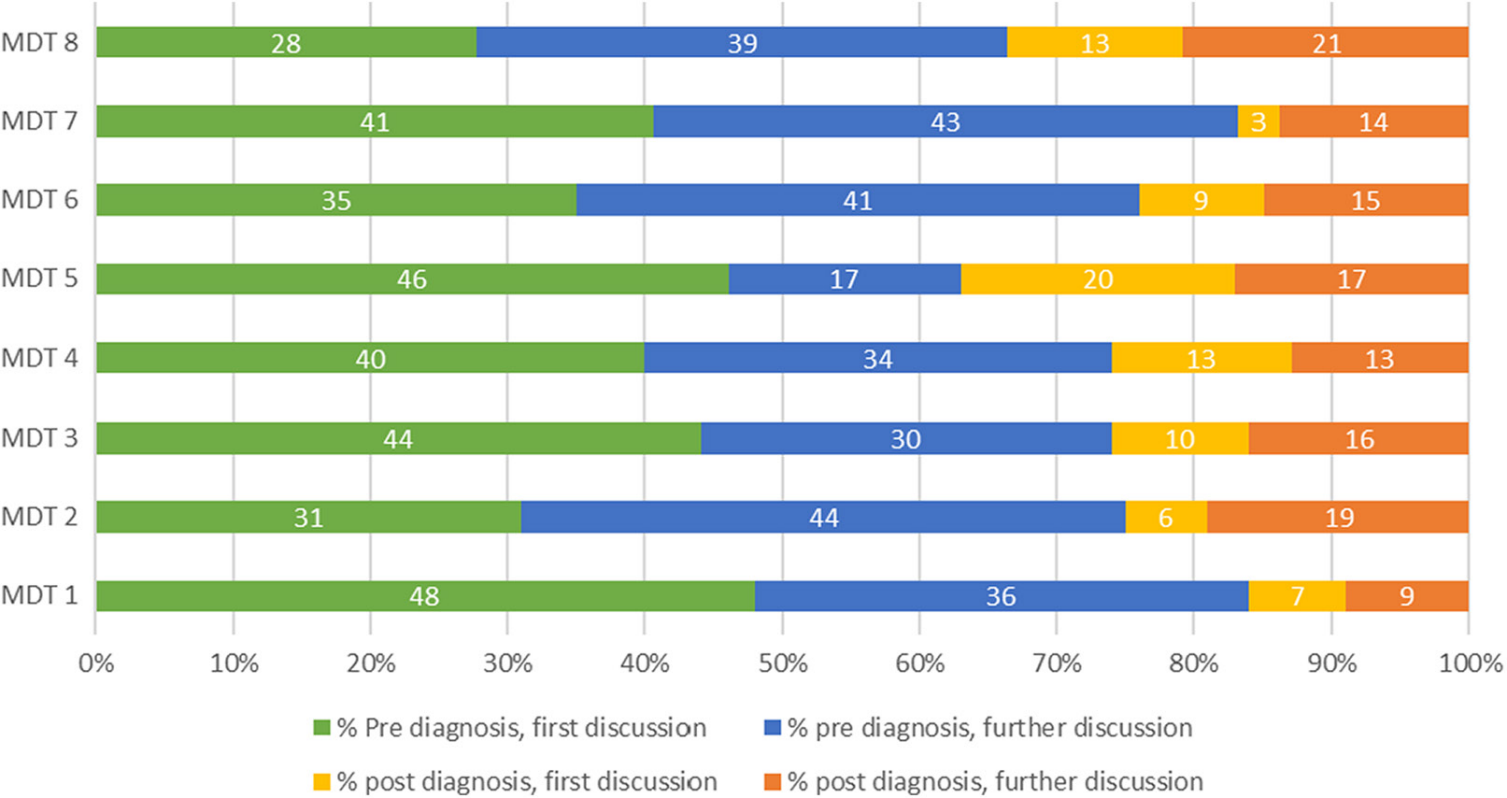
Type of patients discussed in observed MDTMs: Pre‐ versus post‐ diagnosis.

The variability in relation to referral processes, triage methods, and existence of other lung‐related MDTMs or forums within the hospital (in particular whether or not they had a separate diagnostic MDTM)—described above—meant that the types of cases discussed in full MDTMs also varied between the eight MDTs. Regardless of the models of triage or other MDTs/meetings outside of the full MDTM, most cases (71% overall, ranging 58%–76%) listed for discussion in lung MDTMs were prior to lung cancer diagnosis (Figure 1).

More than a third of all cases discussed (37%, range from 17% to 44%) were pre‐diagnostic patients that had been discussed at least once previously in the full MDTM (see Figure 1—pre‐diagnosis—further discussion). The remaining cases discussed in the observed MDTMs were post‐diagnostic cases, that is those with a previously confirmed lung cancer diagnosis (23%, ranging 14%–34%), either soon after initial diagnosis or for a later recurrence. Nine per cent of cases overall were listed for a first discussion post‐diagnosis and 14% had already been discussed post‐diagnosis in a previous MDTM, but within the same episode of care, e.g. for the same recurrence.

In most MDTs, there was a view that some patients were discussed unnecessarily in the full MDTM. A variety of reasons was given for this including that the MDTM provided the only opportunity in some MDTs for a benign case to be discussed with a surgeon, for example where the surgeon worked for a different hospital and was not easily accessible otherwise. Reference was also made to a team culture where it had become the norm to list patients for MDTM discussion, regardless of whether it was appropriate or not, and therefore to have patients discussed multiple times:

That's the culture of how the MDT has worked at [hospital] and how people work here (…) there needs to be a culture change from discussing everybody multiple times before you make any decision about the patient into making decisions that are fairly straightforward outside the MDT [MDT lead]

In some MDTs the availability of slots for diagnostic procedures and the radiology departments' preferred process of booking in patients affected the timeliness of diagnostic results and consequently caused difficulty in implementing diagnostic “bundles” (as recommended in National Optimal Lung Cancer Pathway^16^). This appeared to be one reason why patients were discussed multiple times at MDTMs before the diagnostic pathway was completed:

the radiologists… for example, if we wanted a CT biopsy, they want to see the PET first, and discuss it in the meeting to agree that a CT biopsy is appropriate. (…) it's whether we can persuade them to, or somehow find a way of doing that outside of the meeting [MDT Lead]

In some MDTs, referral to the MDTM appeared to have become an administrative ‘safety net’ to ensure that patients were adequately documented, tracked and kept on the appropriate pathway and respondents indicated that there was varied interest to move away from this way of working:

there is an over‐reliance on the cancer MDT to trigger all the conversations that need to be had about next investigation and treatment [radiologist]

### 3.4 Information available to inform MDTM recommendations

The reason for discussion, case details and radiological information were available for most case discussions in all eight MDTs, but there was considerable variability in relation to the inclusion of most other categories of information (Table 2). Notably, information about the patients' fitness and/or their PS score was available for 60% of cases overall, ranging between 30% and 75% cases by MDT. Availability of information about patients' symptoms, smoking status, comorbidities, pathological information, staging and lung function was similarly variable (Table 2). Patient/family views and psychosocial factors were only available/shared in 12% and 10% of overall cases, respectively.

**TABLE 2:** Information available on agenda or presented in MDTM by team.

Type of information	MDT (%)								
	MDT1	MDT2	MDT3	MDT4	MDT5	MDT6	MDT7	MDT8	All MDTs combined %
Reason for discussion	96	99	99	91	97	98	99	98	**97**
Case details	96	98	98	91	87	84	98	96	**93**
Radiological	96	89	100	98	80	75	97	94	**91**
PS or fitness	75	48	30	72	63	66	71	54	**60**
Symptoms	64	45	55	67	48	25	55	50	**51**
Smoking status	65	37	42	66	39	32	77	37	**49**
Comorbidities	54	45	54	52	32	28	71	54	**48**
Pathological	56	33	61	49	61	40	39	46	**47**
Staging	15	15	45	56	49	18	28	43	**32**
Other cancer history	32	20	27	21	14	21	35	27	**25**
Lung function	28	16	29	15	32	6	43	16	**21**
Patient/Family views	7	7	13	10	21	12	10	18	**12**
Psychosocial	15	10	5	7	6	9	19	9	**10**
Clinical trials	0	0	0	1	0.8	1	0	2	**<1**

The % overall from all 8 MDTs (in bold).

The flow and availability of information was seen as key to the efficient running of MDTMs by core members that were interviewed, in particularly noting the impact of quality and completeness of information at the point of referral to the MDTM. All but one MDT had a proforma (standardised form) for patient referrals to the MDTM, but these were often poorly used/completed; the MDTs depended on GPs and their colleagues in other hospital departments to follow referral procedures and provide comprehensive and accurate information on referrals, but this was often not the case:

we do get the occasional, “This looks like a cancer. Please see them urgently,” and then they just stick it in an envelope [MDT Coordinator]

One MDT coordinator expressed that they felt disempowered when dealing with poor quality referrals:

it's up to the referring clinician to provide the data. I can't go back and say, “That's not there. I'm not listing it”, because I'm not clinical [MDT coordinator]

It was also mentioned that in some MDTs there was insufficient or inadequately trained administrative staff to support collection of the required data/information for each listed case:

my barriers are a lack of admin staff who are specifically trained to deal with lung cancer patients, because our cancer office all revolve and one day we have one co‐ordinator, and another day we have another co‐ordinator [MDT lead]

MDT members judged that they did not have sufficient time to prepare for MDTMs which would include assessing which referrals should be listed for discussion and which sent back for more information and providing comprehensive patient information for MDTM agendas. This was made more time consuming by having to access multiple systems for information:

So, if you have to gather information for a patient who has got a lung cancer, and you need the pulmonary function test, you need the CT, the PET CT but which normally is done by a radiologist. You need the histology, and you need the previous investigations from different places. For one patient that can take you like an hour and a half, two hours already. [surgeon]

This was often perceived as difficult to resolve as the staff time required to prepare for MDTMs would need to be agreed across multiple departments in the hospital, which would not necessarily benefit directly from having a more efficient MDTMs.

### 3.5 Types of MDTM recommendations

Despite being the putative aim of MDTMs, only half (53%) of observed case discussions concluded with a recommendation for treatment (48%) or to discuss treatment options with a patient (5%). This varied across MDTs (see Table 3), being related to the types of cases discussed, and information available in full MDTMs. A third (32%) of discussions resulted in a recommendation for further diagnostics (28%) or to discuss diagnostics with the patient (4%). A significant proportion of case discussions resulted in a decision to defer the case to a subsequent MDTM (24% overall) with variation between MDTs (see Table 3). Delays in diagnostic pathways were a key factor explaining this; in six MDTs the most common reason was due to awaiting results (14% cases overall). In two further, MDTs the most common reason for deferring the case was that the patient required an appointment with an MDT member (7% cases overall), for example to assess their fitness or obtain more information.

**TABLE 3:** MDTM recommendations.

MDTM recommendation^a^	% Cases overall (% Range per MDT)
Treatment of any type	48 (41–61)
Discuss any treatment with patient	5 (2–11)
Diagnostics definite	28 (22–35)
Discuss diagnostics with patient	4 (1–12)
Refer to other cancer MDT or specialist	9 (4–12)
Refer to non‐cancer MDT or specialist	3 (0–7)
Discharge/Not lung cancer	8 (1–14)
Decision deferred	24 (13–36)
No clear outcome	0.6 (0–1)

^a^ Categories are not mutually exclusive.

In all MDTs, inefficiencies in diagnostic pathways were highlighted in interviews as a barrier to effective working and decision making in MDTMs. Diagnostic staffing shortages were cited as the underlying factor for delays in conducting and reporting investigations in most MDTs and some MDTs relied on agreements with other hospitals to provide pathology services.

## 4 DISCUSSION

This mixed methods study of lung cancer MDTMs across a regional network is the largest observational collection of lung cancer MDTM data to date (to our knowledge). It indicates that even within a relatively limited geographical area in the UK, there is considerable variation in terms of MDTM processes and ways of working, particularly the MDTs' structures and processes relating to referral and diagnostic pathway management. These differences are driven by contextual and organisational factors (internal and external), which cannot be easily overcome, implying that streamlining MDTMs and improving efficiency cannot be achieved via a one‐size‐fits‐all approach. Despite this, there are several possible measures which could improve the efficiency of lung MDTMs.

Considerable MDTM time is spent confirming diagnoses with multiple discussions per patient prior to initiating treatment. Data from this study indicates that if clinicians managed a proportion of cases individually or within a smaller team up to the point of diagnosis, it would reduce the number of cases discussed in the MDTM. This would require alterations in clinician job plans to provide adequate time to triage cases referred to the MDTM and to discuss patient diagnostics in other appropriate forums. Adequate governance must exist around patient tracking lists and associated meetings, as it has been previously found that some clinicians are reluctant to streamline due to concerns about the detrimental impact this may have on the quality and safety of patient care, including clinical governance process.^17^ Implementing diagnostic pathways, such as the Diagnostic Standards of Care included in the National Optimal Lung Cancer Pathway,^16^ would speed up the pathway to the treatment decision.

Furthermore, data from this study reinforce the need to prioritise addressing workforce shortages, both in clinical and administrative roles, although it is recognised that the COVID‐19 pandemic placed a substantial burden on staff during the data collection period. This is particularly evident for radiology and pathology expertise,^8^ but inadequate administrative support was also highlighted in some MDTs as providing an obstacle to streamlining MDTM processes and patient pathways. It has been previously shown in international research with MDTs for different tumour types that sufficient protected time for preparing and participating in MDTMs is crucial,^18^, ^19^ including involvement of support staff who can assist clinicians in time consuming tasks such as information preparation and treatment plan monitoring^19^ The potential for improvement regarding internal and external input to MDTMs from pathology, radiology, oncology and surgery also needs to be examined with insufficient pathology and radiology input found to be a barrier to MDT formulating recommendations,^20^ enlisting local, regional and national support for increased staffing levels and time dedicated to supporting diagnostic pathways.

A large proportion of MDTM case discussions did not include key clinical information about the patient. Although use of MDT referral proformas has been recommended to ensure information is available to the MDT^7^ and most of the MDTs involved in this study had a proforma available, these varied considerably in terms of the information requested and in relation to their uptake and use. The forms were often incomplete, or not submitted. Improved uptake could be achieved by educating internal and external referrers about the MDT's needs and expectations, for example by sending back incomplete referrals with a request for further information prior to the MDTM, however this is a resource intensive process that may, in the short term, be detrimental to patient care. Studies of MDTs for different tumour types within the UK^21^ and internationally^22^, ^23^, ^24^ found that the quality of information provided in meetings impacts the likelihood of a decision being made and this also varied in the observed MDTMs. Agreeing a minimum dataset for a case to be discussed in a MDTM, as developed for lung MDTs in Australia,^23^ and improving the transfer of this information, via MDTM agenda or case presentation, is likely to support more efficient and effective decision‐making in MDTMs. The data presented here are part of a wider study that used a modified Delphi process to develop shared consensus on protocols for MDT working and minimum datasets for referral (Harris et al, paper in preparation, data available on request).

## 5 CONCLUSION

This study highlights the complexity of factors influencing the ways that lung MDTs in the UK are structured and the differing ways in which they work. Improving efficiency of the MDT pathway will therefore require contextualised approaches that take into account these differences. However, based on our findings we recommend that MDTs: (1) work with stakeholders to standardise the referral process and improve the quality of clinical information on referrals; (2) commit adequate clinical and administrative resources to support managing caseloads more effectively outside of MDTMs; and (3) examine potential for improvement regarding internal and external input from pathology, oncology and surgery. Finally, local, regional and national support are needed for lung MDTs to assist them in developing their diagnostic workforce and facilities. The findings from this research have implications for cancer multidisciplinary working in a variety of contexts, with similar challenges to efficient MDTMs noted in research outside of the UK and in other tumour types.

## AUTHOR CONTRIBUTIONS

**Magdalena Zasada:** Formal analysis (supporting); investigation (equal); project administration (equal); writing – original draft (lead); writing – review and editing (lead). **Jenny Harris:** Formal analysis (lead); methodology (equal); writing – review and editing (equal). **Johanna Groothuizen:** Investigation (equal); project administration (equal); writing – review and editing (equal). **Eunice Aroyewun:** Investigation (equal); project administration (equal); writing – review and editing (equal). **Jeewaka Mendis:** Formal analysis (supporting); methodology (equal); writing – review and editing (equal). **Cath Taylor:** Conceptualization (equal); funding acquisition (equal); methodology (equal); project administration (equal); supervision (equal); writing – review and editing (equal). **Madeleine Hewish:** Conceptualization (equal); funding acquisition (equal); methodology (equal); supervision (equal); writing – review and editing (equal).

## CONFLICT OF INTEREST STATEMENT

All authors have declared there are no financial conflicts of interest with regards to this work.

## ETHICS APPROVAL STATEMENT

This study was reviewed and approved by the UK Health Research Authority (IRAS 270697) following approval from the NHS London Fulham Research Ethics Committee (ref. 19/LO/1699) and Confidentiality Advisory Group (ref. 19/CAG/0211). Study researchers held: (1) Fully completed and signed Research passport document; (2) Letter of Access from each participating organisation; (3) Additional confidentiality agreements as required by individual organisations depending on their own requirements for CAG supported studies. Participants consented to the study by returning informed consent forms.

## Supporting information


Data S1.

## Data Availability

The data that support the findings of this study are available upon reasonable request from the corresponding author. The data are not publicly available due to privacy or ethical restrictions.
