# Supplementary material for: Investigating the efficiency of lung multi‐disciplinary team meetings—A mixed methods study of eight lung multi‐disciplinary teams
Source: Cancer Med. 2023 Mar 19;12(8):9999–10007. doi: 10.1002/cam4.5730 (PMC10166965; doi:10.1002/cam4.5730)
Supplement: Supplementary file 1 — Data S1. [file CAM4-12-9999-s001.docx]

**Items included in the modified MDT-QuIC form**

| **Item** | **Categories (options for researchers to select on QuIC form)** | **Data source** |
| --- | --- | --- |
| **Meeting attendance (collected for each core MDT member)** | Full meeting attended  Partial attendance – absent for >15 minutes  Partial – absent for 5-15 minutes  Partial – absent for <5 minutes at start or end  Intermittent attendance  Absent from meeting | Live in MDTM |
| **Information presented in meeting** | Case details  Symptoms  Reason MDT discussion  Radiological  Pathological  Staging  Other cancer history  Comorbidities  Smoking status  PS score  Fitness  Lung function  Herder/Brock score  Psycho-social  Patient/family views  Clinical trials | Live in MDTM |
| **Information available on agenda** | Case details  Symptoms  Reason MDT discussion  Radiological  Pathological  Staging  Other cancer history  Comorbidities  Smoking status  PS score  Fitness  Lung function  Psycho-social  Patient/family views  Clinical trials | MDTM agenda |
| **Outcome** | Treatment recommendation/decision not to treat  Discussion of defined treatment options with patient  Further diagnostics  Discussion of defined diagnostic options with patient  Referred to another cancer MDT/specialist  Referred to non-cancer specialist  Not lung cancer/discharged  Decision deferred – awaiting results  Decision deferred – MDT member missing/needed  Decision deferred – appointment with MDT member needed to assess/discuss further steps  Decision deferred – information missing | Live in MDTM |
| **Type of patient at time of referral to meeting** | Pre-diagnosis, first discussion  Pre-diagnosis, further discussion  Post-diagnosis, first discussion  Post-diagnosis, further discussion | Somerset Cancer Register |
